# Drug-Eluting Sandwich Hydrogel Lenses Based on Microchamber Film Drug Encapsulation

**DOI:** 10.1021/acsnanoscienceau.2c00066

**Published:** 2023-04-05

**Authors:** Valeriya Kudryavtseva, Mariana Otero, Jiaxin Zhang, Anton Bukatin, David Gould, Gleb B. Sukhorukov

**Affiliations:** † School of Engineering and Materials Science, Queen Mary University of London, London E1 4NS, U.K.; ‡ National Research Tomsk Polytechnic University, 30 Lenin Avenue, Tomsk 634050, Russian Federation; § Biochemical Pharmacology, William Harvey Research Institute, Queen Mary University of London, London EC1M 6BQ, U.K.; ∥ Alferov Saint Petersburg National Research Academic University of the Russian Academy of Sciences, 8/3A Khlopina str., Saint Petersburg 194021, Russian Federation; ⊥ Institute for Analytical Instrumentation of the Russian Academy of Sciences, 31-33 A, Ivana Chernykh str., Saint Petersburg 198095, Russia; # Skolkovo Institute of Science and Technology, Bolshoy Boulevard 30, bld. 1, Moscow 121205, Russian Federation; ∇ Siberian State Medical University, Moskovskiy Trakt, 2, Tomsk 634050, Russian Federation

**Keywords:** drug delivery system, dexamethasone, drug-eluting contact lens, extended-wear contact lens, polylactic acid, soft lithography

## Abstract

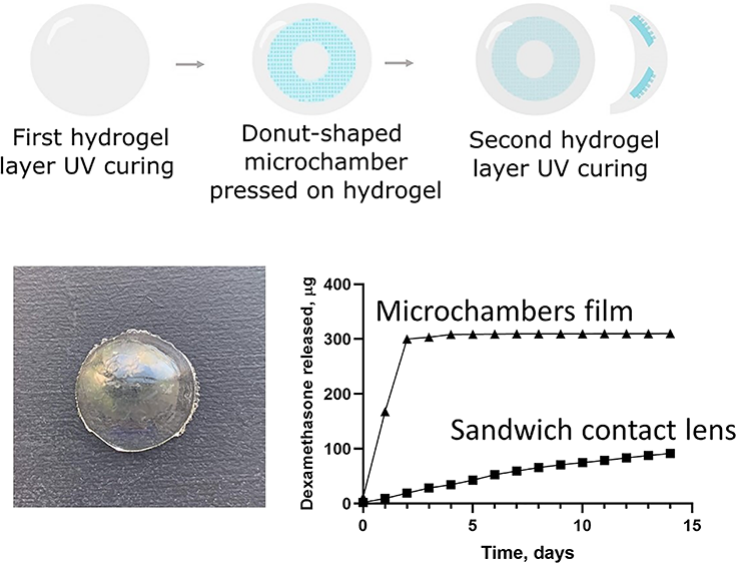

Corticosteroids are widely used as an anti-inflammatory treatment for eye inflammation, but the current methods used in clinical practice for delivery are in the form of eye drops which is usually complicated for patients or ineffective. This results in an increase in the risk of detrimental side effects. In this study, we demonstrated proof-of-concept research for the development of a contact lens-based delivery system. The sandwich hydrogel contact lens consists of a polymer microchamber film made via soft lithography with an encapsulated corticosteroid, in this case, dexamethasone, located inside the contact lens. The developed delivery system showed sustained and controlled release of the drug. The central visual part of the lenses was cleared from the polylactic acid microchamber in order to maintain a clean central aperture similar to the cosmetic-colored hydrogel contact lenses.

## Introduction

Eye drop solutions are widely used to treat eye diseases. However, eye drops are associated with many limitations. For instance, for noninfectious ocular inflammatory diseases, such as uveitis, eye drops are administered as often as once every hour. The ocular bioavailability of eye drops is less than 5%, and the rest of the drug enters the bloodstream via a conjunctival or nasal path which can cause severe side effects.^1^ The development of new targeted drug delivery systems for ocular drugs aims to improve their bioavailability.

Usually, drug administration through eye drops results in a burst delivery, which necessitates the delivery of multiple drops each day.^2^ Sustained drug delivery can be achieved using a variety of delivery systems including implants, microparticles, nanoparticles, and gels or their combination. Many novel ophthalmic drug delivery systems have been proposed including—mucoadhesive films, in situ gels,^3^ liposomes,^4^ microemulsions,^5^ polymeric nanoparticles, collagen shields, rods, inserts, rings, etc.

Drug-eluting coated contact lenses can be a potentially better alternative to long-term eye drug delivery devices as a convenient and patient-friendly approach for drug administration. Contact lenses recently have been receiving a lot of attention as a medical device for controlled ophthalmic drug delivery.^6^ The use of contact lenses as a drug delivery device allows a dose reduction, along with a decrease in nonproductive systemic drug absorption and associated side effects.^7,8^ However, loading the sufficient drug into contact lenses and controlling the release of the drug are still challenging.

Many methods have been reported for sustaining the drug delivery using contact lenses—soaking method, molecular imprinting, entrapment of drug-loaded colloidal nanoparticles or film, and supercritical fluid technology, which were extensively reviewed recently by Xu et al.^9^ and Rykowska et al.^10^ Each of the proposed methods is associated with some limitations. For example, traditional soaking methods show low drug loading and rapid diffusion within hours.^11^ In addition, drugs with low solubility cannot be loaded using this method. An approach based on the vitamin E diffusion barrier is associated with changes in the mechanical properties and absorption of the protein due to its hydrophobic nature.^12^ In molecular imprinting, drug loading is limited by functional monomers and template molecules. Moreover, the highly cross-linked structure of the hydrogel can affect the optical and mechanical properties of contact lenses.^12^ Other problems associated with therapeutic contact lenses include drug stability during processing and manufacturing, burst release of the drug, and drug loss due to sterilization and during storage.^13^

To overcome these limitations, different approaches are being proposed, including sandwich-like contact lenses with different combinations of drugs, materials, and methods of encapsulation. A new type of ocular drug system based on hydrogel contact lenses has been proposed by Ciolino’s group.^14^ It was shown that such a DDS can provide sustained drug release at therapeutic rates without alerting the optical and physical properties of the lenses. Another approach was proposed by Pimenta et al. based on triple-layer contact lenses, where a drug-loaded middle layer is sandwiched by drug-free outer layers. It was shown that such a construct can be applied to suppress the burst release of the cargo.^15^ An example of similar sandwich technology was shown by Maulvi et al.^16^ They proposed an ethyl cellulose nanoparticle-laden ring incorporated into hydrogel contact lenses. Later, the same group developed a similar ring system,^17^ where the contact lenses were equipped with HA-laden ring implants for sustained ophthalmic drug delivery. According to the authors, such a system did not affect the optical and physical properties of contact lenses.

Such contact lenses must be transparent and optically stable for a long period while having a low modulus of elasticity, being hydrophilic at the surface, and being permeable for ions and oxygen to allow normal corneal metabolism and respiration during lens wearing.^18^ The average size of normal pupil diameter in adults ranges from 2 to 4 mm in bright light and 4 to 8 mm in the dark; thus a central zone of at least 5 mm diameter has to be transparent for clear visual function.^19^ The recently developed drug delivery strategy based on drug encapsulation in an array of polymer microchambers seems to be a suitable option for this purpose.^20^ The microchamber shell could be made with different types of polymers, including biodegradable polyesters^21,22^ or polyelectrolyte multilayers.^23−25^ The microchamber approach provides a suitable tool to encapsulate a wide range of substances with almost no limitations, including both soluble and poorly soluble drugs. In this work, we combined two approaches—microchambers drug delivery system and sandwich lenses. Such a system would allow or encapsulation of virtually most drugs envisaged used in ophthalmology and its zero-order release kinetics with maintaining visual function. The microchamber film located between two hydrogel layers contains dexamethasone powder. By adjusting the size of the film and the shape of the microchambers, the drug release can be further regulated. This method can be extended to a wide range of drugs since there is minimal interaction with solvents. The combination of different polymers and ophthalmological drugs offers the potential for microchambers to be used in the development of drug-eluting contact lenses. For instance, the technology can enable the delivery of multiple drugs with specific release times for each component in the system.

## Materials and Methods

### Materials

Polylactic acid (PLA, 3 mm granule, *M*_w_ 193.3 kg/mol) was purchased from GoodFellow; chloroform and dexamethasone (Pharmaceutical Secondary Standard, Supleco) were purchased from Sigma-Aldrich. A poly(dimethylsiloxane) (PDMS) kit from Sylgard was used to create the PDMS stamps. The PDMS kit (Sylgard 184, Dow-Corning, USA) was used for PDMS stamp fabrication by the standard soft lithography technique using a silica mold.^29^ The mold contained an array of 11 × 11 × 22 μm rectangular pillars on an area 20 × 20 mm^2^. It was fabricated by cryogenic reactive ion etching of a silica wafer with a chromium mask made by lift-off lithography. 2-Hydroxyethyl methacrylate (HEMA) (97%, Sigma-Aldrich), ethylene glycol dimethacrylate (EGDMA) (98%, Sigma-Aldrich), and photoinitiator (Irgacure 2959; Ciba) were used for the hydrogel solution. Phosphate buffer saline (PBS) (tablet, Sigma-Aldrich) and sterilized water (Sigma-Aldrich) were used to make the PBS solution. To label the samples for confocal microscopy, Nile red (Sigma-Aldrich), 5(6)-carboxyfluorescein (CF) (Sigma-Aldrich), and dexamethasone fluorescein (ThermoFisher Scientific) were used.

### Microchamber Fabrication and Dexamethasone Encapsulation

**Figure 1:**
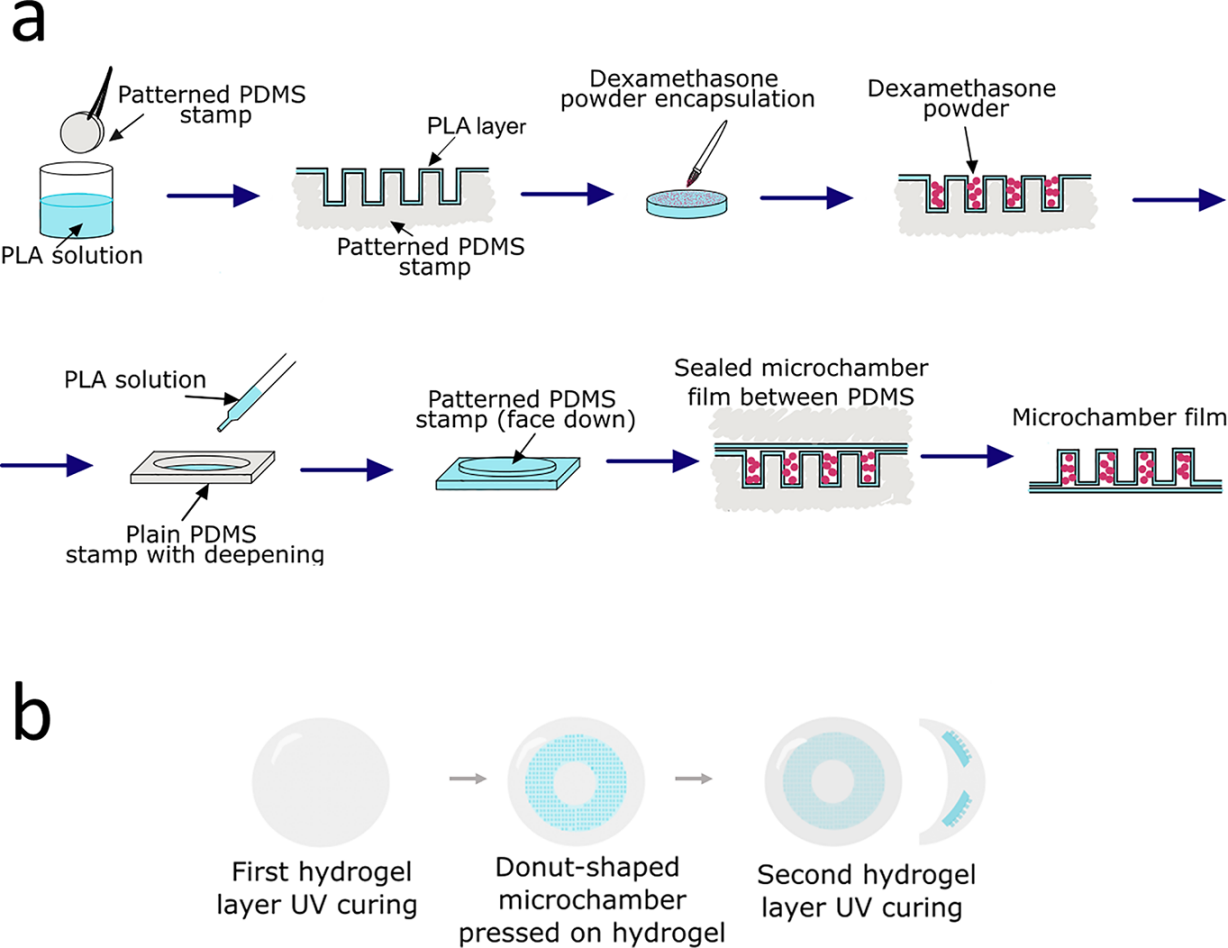
Schematic illustrations of (a) free-standing microchambers fabrication process and (b) drug-eluting hydrogel lens fabrication protocol.

Prior to microchamber fabrication, a Precellys 24 homogenizer was used to mill dexamethasone powders before encapsulation. The dexamethasone was milled for 3 min at 6800 rpm for 30 s with 60 s pauses. The powder particle size was determined based on three images captured from different fields of view using the ImageJ 1.53t software and at least 50 measurements. The microchamber fabrication process is illustrated in Figure 1a. For the fabrication of the microchamber free-standing film, first, a patterned PDMS stamp as a negative replica of the silica master stamp was prepared. The PDMS stamp was dipped into 2 wt % PLA solution for 5 s to coat the stamp with a thin polymer layer and left for solvent evaporation in ambient conditions. After that, the dexamethasone milled powder was distributed with a soft brush on top of the patterned PDMS stamp covered with a thin polymer layer. Second, a flat PDMS stamp with a circular indent was prepared to seal the microchambers. Three hundred microliters of PLA solution was pipetted into the flat PDMS stamp, to form a thick layer of PLA. The chloroform was left to evaporate, and then the flat PDMS stamp was dipped into the PLA solution once again to have a sticky polymer layer on top. The patterned PDMS containing the encapsulated microchambers was placed onto the flat PDMS to seal the microchambers. The PDMS stamps were pressed together using a force of approximately 5 N for a period of 5–7 min. The PMDS stamps were then detached, leaving the sealed microchambers on the patterned PDMS. The attached PDMS stamps were left to dry for 2 h inside a vacuum chamber. After that, the patterned PDMS stamp was carefully removed, and the free-standing film was detached using tweezers. An 8 mm diameter biopsy knife (Integra) was used to cut out a hole in the center of the sample to form a doughnut shape. Finally, the microchamber films were left in sterile water for 2 h to remove unsealed dexamethasone and were stored in sterile Petri dishes. The thickness of the microchamber shell was calculated based on the scanning electron microscopy (SEM) images of broken microchambers.

### Hydrogel Lens Fabrication

To prepare hydrogel sandwich lenses similarly to that in ref (26), 44 μL of ethylene glycol dimethacrylate (EGDMA) was added into 11.6 mL of HEMA monomer followed by 100 μL of 0.1 g/mL photoinitiator (Irgacure 2959, Ciba) in dimethyl sulfoxide. Three hundred and fifty microliters of the resulting solution were transferred into a custom-made female mold. The solution was polymerized with a 305 nm UV lamp in UVACUBE 100 for 50 min to form the bottom pHEMA layer of the composite contact lens. The microchamber was manually lightly pressed onto the pHEMA gel, after that another 350 μL of monomer photoinitiator solution was added and left for UV polymerization for 50 min. The resultant contact lens prototype was a sandwich-like lens with a thin donut-shaped PLA microchamber film with encapsulated dexamethasone coated with pHEMA on both sides. The deposition of the microchamber was out of the visual area (Figure 1b).

### Scanning Electron Microscopy

SEM (ESEM Quanta 400 FEG, FEI, USA) was used to study the morphology of obtained samples with imaging conditions of 10 kV accelerating voltage and 10 mm working distance. Before the investigation, the material surface was coated with a thin gold layer (Agar Auto Sputter Coater, Agar Scientific, UK). For the imaging of the cross section of the lens, hydrated samples were cut with a scalpel surgical blade and placed on the side on the pin stubs covered with adhesive carbon tabs.

### Confocal Laser Scanning Microscopy (CLSM)

CLSM (ZEISS LSM710, Germany) was used for microchamber characterization. The polymer shell was labeled with Nile Red. To label PLA, about 1 mg of Nile Red powder was added to 20 mL 2 wt % PLA solution. Dexamethasone-FITC, a green fluorescent analogue of dexamethasone, was used as a cargo. Two lasers were used to view the different components with an excitation of 543 nm to visualize the microchamber shell and an excitation of 488 nm to visualize dexamethasone-FITC, respectively. Obtained images were processed using the ZEISS ZEN software.

### Dexamethasone Release Measurements

For release, the samples were placed in 5 mL of cell media and incubated at 37 °C for the duration of the experiment without stirring. Every 24 h, probes of the solutions the samples were removed to be tested and the samples were placed in fresh 5 mL media. The mass of the dry sandwich lens was 0.426.9 ± 47.9 mg. The experiment ran for 14 days. The cell media were made by adding 500 mL of Dulbecco’s modified Eagle’s medium (high glucose, Sigma-Aldrich), 50 mL of inactivated serum, and 5 mL of penicillin streptomycin glutamine (Gibco by Life Technologies). A reporter cell assay for glucocorticoids was used to monitor the daily dexamethasone release as described by Read et al.^27^ All samples were measured in triplicate.

### IVIS Optical Imaging

To visualize drug distribution inside hydrogel lenses during the incubation and release experiment, an IVIS Lumina III Fluorescence and Bioluminescence instrument was used. Sandwich lenses with microchamber films with encapsulated dexamethasone-FITC and CF were prepared. Samples were incubated in 0.01 M PBS solution at 37 °C. Every 24 h, samples were placed in a new PBS solution. Empty hydrogel lens and sandwich hydrogel with empty microchambers were used as controls.

### Transmittance

A PerkinElmer Lambda35 UV–vis spectrometer was used to measure the light transmission through hydrated samples. Hydrated contact lenses were placed in a sample holder the way the beam was positioned in the center of the lens. Light transmission was calculated by averaging the transmission over the visible light spectrum (400–700 nm). Samples were incubated in 0.01 M PBS solution at 37 °C. Every 24 h, samples were placed in new PBS solution. The experiment ran for 14 days. The light transmission of empty lens samples and sandwich lens samples with encapsulated dexamethasone was compared. Samples were measured in triplicate.

### Statistics

Statistical analyses were performed in GraphPad Prism, version 9.4.1 for Windows (GraphPad Software, USA) using the unpaired *t*-test and one-way analysis of variance. All results are presented as mean ± SD.

## Results and Discussion

### Sandwich Lens Fabrication of Drug Encapsulation

Dexamethasone is a corticosteroid drug with low solubility in water widely used for the treatment of various inflammatory conditions including ophthalmological disease. In the case of eye treatment, corticosteroids present a few issues such as poor penetration of the cornea, short duration of action, and poor absorption of the drug. The general procedure of taking the drug requires the use of high concentrations of the substance as well as repeated dosage leading to adverse side effects.^28^ Therefore, dexamethasone was chosen as a model drug for sandwich-like lens demonstration.

**Figure 2:**
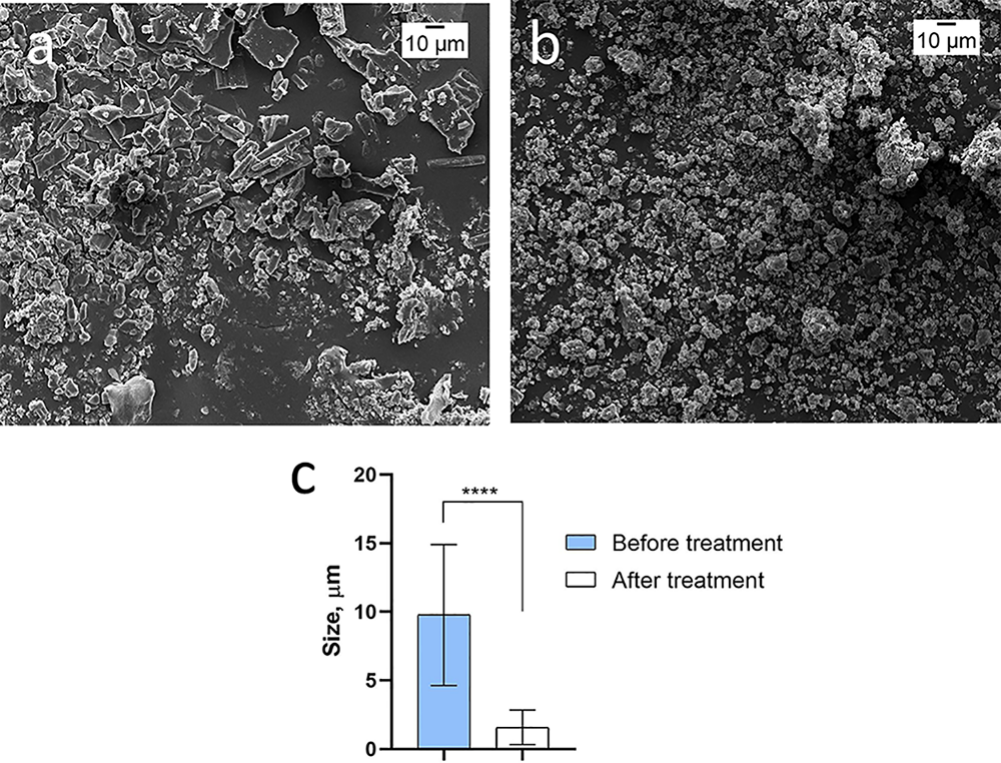
SEM images of dexamethasone powder (a) untreated, (b) milled for 3 min with homogenizer at the same magnification, and (c) dexamethasone powder particle size before and after treatment with a homogenizer, ****p < 0.0001, unpaired *t*-test.

The fabrication process is based on a soft-lithography method. The free-standing microchamber film fabrication is a multistep process that has been previously described.^21,29−32^ The schematic illustration is presented in Figure 1. Due to dexamethasone low solubility (10 mg/100 mL), it was encapsulated from the dry powder. Previously, a dry-loading encapsulation method for microchambers was suggested by Zhang,^22^ and this method can be used for most types of cargo agents as the solubility no longer needs to be considered. Such a method offers a very effective encapsulation when the powder is distributed on the sample surface with a soft brush. However, in the case of encapsulation of cargo from the powder, the particles may be bigger than the size of the well in the PDMS stamp. To overcome that and increase encapsulation rate and payload, particles can be milled to reduce their size. Dexamethasone untreated powder particles had a size up to 20–25 μm with a mean size of 9.7 μm. After they were milled with a homogenizer, the size of the crystals decreased up to a submicron, with a mean size of about 1.6um, making them appropriate for loading into the microchambers (Figure 2).

**Figure 3:**
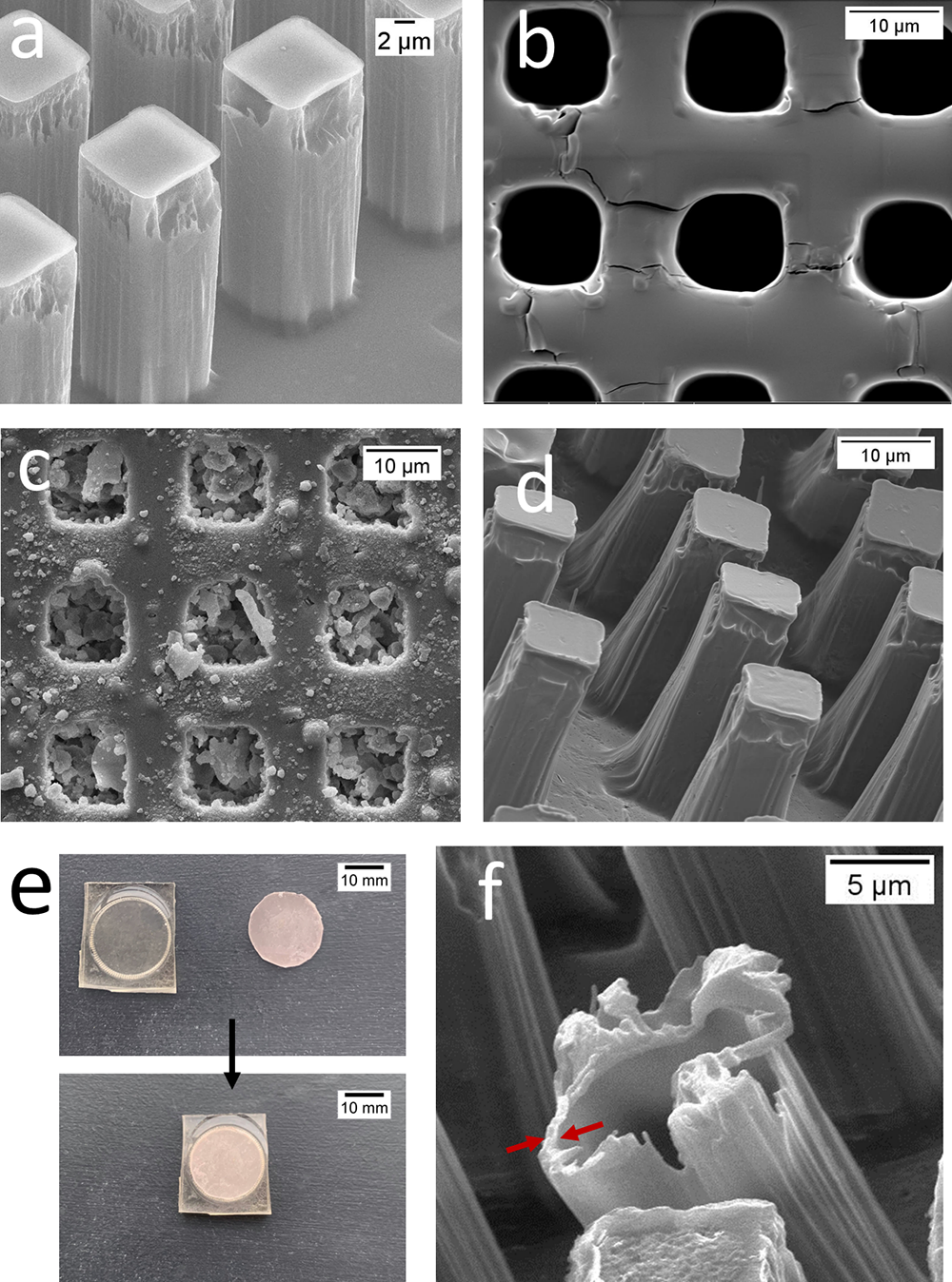
SEM images of (a) silica master stamp, (b) PDMS stamp, (c) dexamethasone powder particles encapsulated in PDMS covered with a PLA thin layer, (d) microchamber free-standing film, and (e) PDMS stamps for fabrication of the donut-shaped microchamber film. Photo of the circular indented PDMS stamp, plain PDMS stamp and circular indented PDMS stamp, and plain PDMS stamp together; (f) SEM image of broken microchambers; red arrows indicate the thickness of the microchamber polymer shell.

The shape of microchambers is important as it affects both film mechanical properties and encapsulation and release control. In our study, we used 11 × 11 × 22 μm cuboid-shaped chambers as done by soft lithography. The SEM image of the silica master stamp is shown in Figure 3a, and the corresponding PDMS stamp is in Figure 3b. The chosen shape allows for easy encapsulation of high amounts of cargo compared to other methods due to its high aspect ratio and the size of the wells being bigger than most of the dexamethasone particles (Figure 3c). This technique allows for the encapsulation of most types of drugs, including low-soluble and sensitive drugs such as antibiotics or proteins. With respect to sensitive drugs, this encapsulation method provides very little direct drug-to-solvent contact, limited only to a few particles on the top of the wells, therefore ensuring that most of the drug inside the well remains intact.

Another important aspect is the choice of polymer. According to previous work,^21^ the particular choice of polymer can significantly affect the shape of microchambers due to different mechanical properties and its rheological properties. PLA was shown to retain cargo better compared to the other biodegradable polymers such as PLGA or PCL.^21^ The molecular weight of the polymer can also affect both preparation of the microchambers and resulting samples, as it can significantly affect the rheological properties of polymer solution and polymer degradation kinetics.^33,34^

The concentration of PLA can also have a significant effect on the samples. Different PLA concentrations result in varying thickness of the microchamber walls, a study by Gai et al.^35^ showed that the thickness of the PLA film increases with increased PLA concentration. Changing the wall thickness can have several consequences on the overall structure of the microchambers. The first is that increasing the wall thickness has an impact on the interior volume of the microchambers. This could lead to a lack of space in which to place the dexamethasone. However, decreasing wall thickness can have a direct effect on mechanical properties and cause an increase in the fragility of the structure which could encourage the overall structure of the microchamber to be damaged. The SEM image in Figure 3d shows that PLA microchambers made with 2% PLA solution can retain their structure upon removal of the film from the patterned PDMS stamp and also have a large enough internal volume for successful drug encapsulation.

Figure 3f shows the broken microchambers from which the thickness of the polymer shell can be estimated. The mean thickness of the wall was calculated to be about 1 ± 0.3 μm, which is the same as in our previous studies.^29^

**Figure 4:**
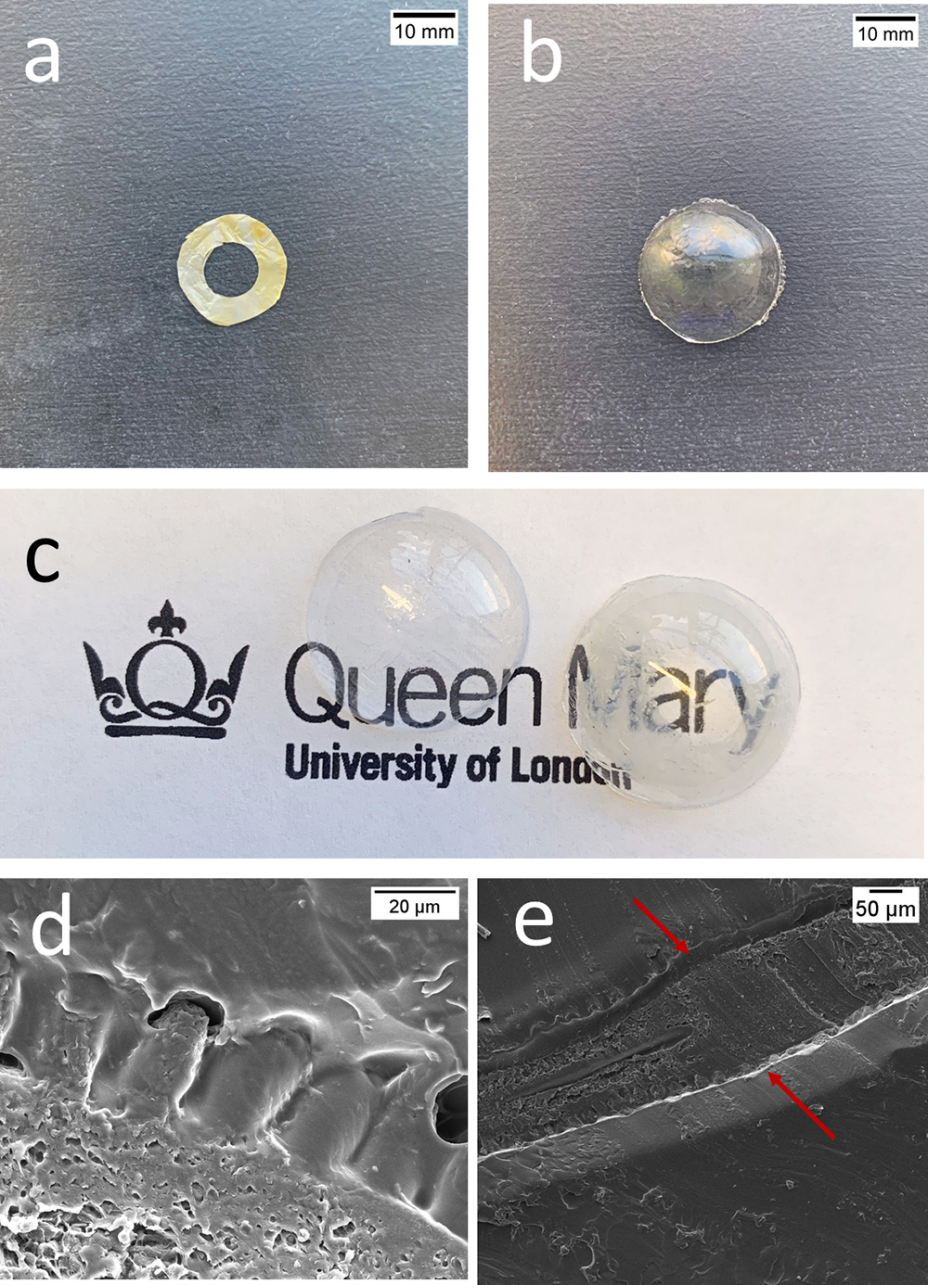
Photo of (a) donut-shaped microchamber film with encapsulated DEX-FITC, (b) sandwich pHEMA lens with microchambers with encapsulated DEX-FITC, and (c) empty pHEMA lens and sandwich pHEMA lens with microchambers with encapsulated dexamethasone (Queen Mary University of London logo used with permission), (d, e) SEM images of the cross section of sandwich pHEMA lens. Red arrows indicate the microchamber film located between two hydrogel layers.

In order to prepare a free-standing film, we used a circular template presented in Figure 3e. The prepared film was cut out in the middle with an 8 mm diameter biopsy pouch knife and sandwich-like lenses prepared as shown in Figure 4a. The size of the free-standing film was 1.9 cm in diameter with an 8 mm diameter cut-out in the center and the overall area equaling 2.2 cm^2^. The total thickness of the free-standing film including the 20 μm tall microchambers is approximately 92 μm. The fabrication method allows for variation in the size of the microchamber film, which in turn can provide variation in the amount of drug that can be encapsulated. An example of a smaller microchamber film is shown in Supporting Figures 1 and 2. The central part of the film was cut out to maintain a clear central aperture similar to that of cosmetic colored hydrogel contact lenses (Figure 4c).

For hydrogel lens fabrication, pHEMA was chosen as it is widely used for hydrogel lens fabrication. The scheme of drug-eluting lenses is shown in Figure 2. The total thickness of the lens was about 1200 μm and a 2.5 cm outside diameter (Figure 4b). The lens and size thickness could be later adapted with more sophisticated manufacturing equipment to become closer to the commercially available lens dimensions which are more comfortable for patients.

The SEM image of the sandwich lens cross section shows how microchambers are located inside the sandwich hydrogel lens (Figure 4d,e). Figure 4d shows that the sample retains its shape between two hydrogel layers.

### Dexamethasone Release Studies

**Figure 5:**
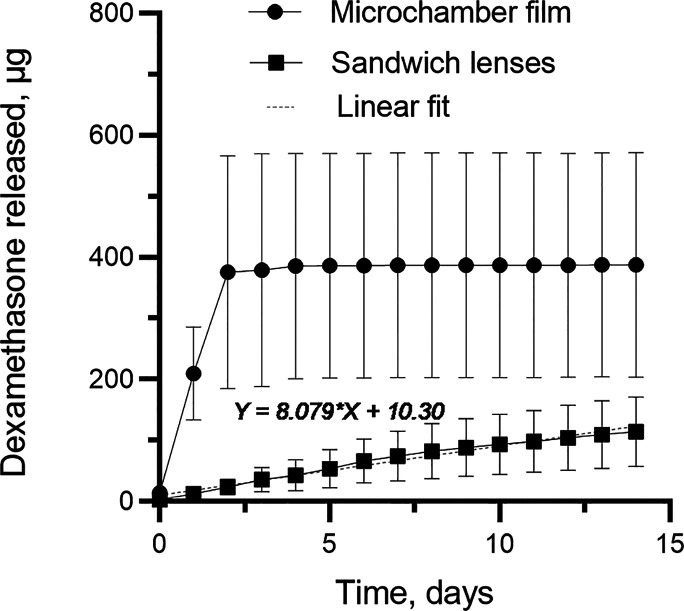
Release profile of dexamethasone from microchambers and drug-eluting lenses during incubation cell media at 37 °C measured by the glucocorticoid sensitive reporter cell line. All values plotted as mean ± SD. The release from sandwich lenses fitted with simple linear regression, *p* < 0.0001.

Dexamethasone can be detected using a glucocorticoid-sensitive reporter cell line, as described by Read et al.^27^ In most studies, steroid release is measured with high-performance liquid chromatography (HPLC) or UV–Vis absorbance methods. HPLC has a reported quantification limit of approximately 10 nM, while UV/Vis has a lower detection limit of around 1 μM. The reported method is not only more sensitive, but also it can be used to validate the biological activity of the drug. Hence, this approach offers a distinct advantage in measuring the drug release from DDSs, as it accounts for the influence of other degradation products, such as hydrogel or degraded polymer, that may compromise the accuracy of the results. Furthermore, it also considers the potential impact of the use of chloroform for microchamber film preparation or UV curing step for hydrogel fabrication, which could potentially affect the biological activity of dexamethasone.^27^ The release profile of dexamethasone from microchambers and drug-eluting lenses is shown in Figure 5.

The extended-wear contact lenses can be worn continually for periods from 1 to 4 weeks without even taking the lenses off at night. Prolonged contact lens wear is associated with the risk of contamination and eye infection.^36^ Therefore, the release was measured only for 2 weeks. The results show that microchamber films have a high release peak compared to the contact lens sample at the beginning of the experiment. The rapid dexamethasone mechanism of release from microchambers could be associated with polymer film defects, degradation of polymer, diffusion, or a combination thereof. Moreover, the geometry of the microchambers also has a significant effect on the release profile of the encapsulated drug. Meanwhile, the contact lens sample showed constant release over the course of the 2 weeks of the study. The results show that dexamethasone was released more slowly from the sandwich-like drug-eluting lens than from free-standing PLA microchambers. This may be due to dexamethasone redistribution inside hydrogel materials which slows down the release. Such a structure gives a more linear release of dexamethasone, which is more favorable for ocular drug delivery. The most likely mechanism of release from sandwich lenses is a diffusion of dexamethasone out of the microchamber film and subsequent slower release from the pHEMA hydrogel.

Typically, dexamethasone is prescribed for 2 weeks in 0.1% ophthalmic solution from 4 times a day to two drops every hour, resulting in about 80–1500 μg a day. Our results show a steady release of dexamethasone of about 100 μg per week, while the total amount of dexamethasone according to release from microchambers is about 400 μg (387.5 ± 125.9 μg). Therefore, the total loading amount of the drug in each microchamber is about 650 pg. (664.5 ± 215.9 pg). The release rate can be increased in a few ways. First, polymers with a higher release rate could be used such as polycaprolactone (PCL) or poly(lactic-*co*-glycolic acid) (PLGA). It was previously shown by a number of studies that microchamber films made of PCL or PLGA can release cargo faster compared to PLA.^21,22^ Another way is to increase the size of the microchamber film and volume of each microchamber by using a different master stamp with pillars. Moreover, Ross et al.^37^ showed that rabbits wearing contact lens dexamethasone drug delivery system achieved 200 times higher retinal drug concentrations compared to hourly dexamethasone drops. Therefore, such sustained delivery of dexamethasone might be enough to treat certain eye conditions without excessive systematic dexamethasone delivery.

The contact lens with a dexamethasone-polymer film developed by the Ciolino group^37^ showed that the total amount of encapsulated dexamethasone was about 1500 released μg and it was fully released in 1 week. Similarly, the layered chitosan-based contact lens developed by Gade et al.^38^ had about 1500 μg of dexamethasone, but it was released just within 6 h. The silicone hydrogel contact lenses formulated with 650 μg dexamethasone released the drug for 60 days.^39^ Typically soaking and vitamin E-assisted encapsulation show much lower encapsulation rates in the range of dozens of micrograms and relatively fast release for about 1 day^40^ according to Kim at al.,^41^ which was extended for about over 1 week by incorporation of Vitamin E into the contact lenses.

The amount of dexamethasone encapsulated in the microchamber film is lower than that suggested by Ross et al.^37^ or Gade et al.^38^ Nonetheless, the developed sandwich contact lenses show zero-order release which can be adjusted by the change in the size of the film and the shape of the microchambers. Due to the drug’s minimal interaction with solvents, this approach has the potential to be applied to a diverse range of drugs, including sensitive molecules such as peptides and proteins. Furthermore, this technology offers the advantage of combining various polymers and ophthalmic drugs, including multiple drugs with specific release times for each component within the system. Another interesting aspect of the developed device is that the anisotropic shape of the microchamber film and its orientation in the lens might result in varying release rates on either side of the lens due to significant differences in the surface area on different sides.

**Figure 6:**
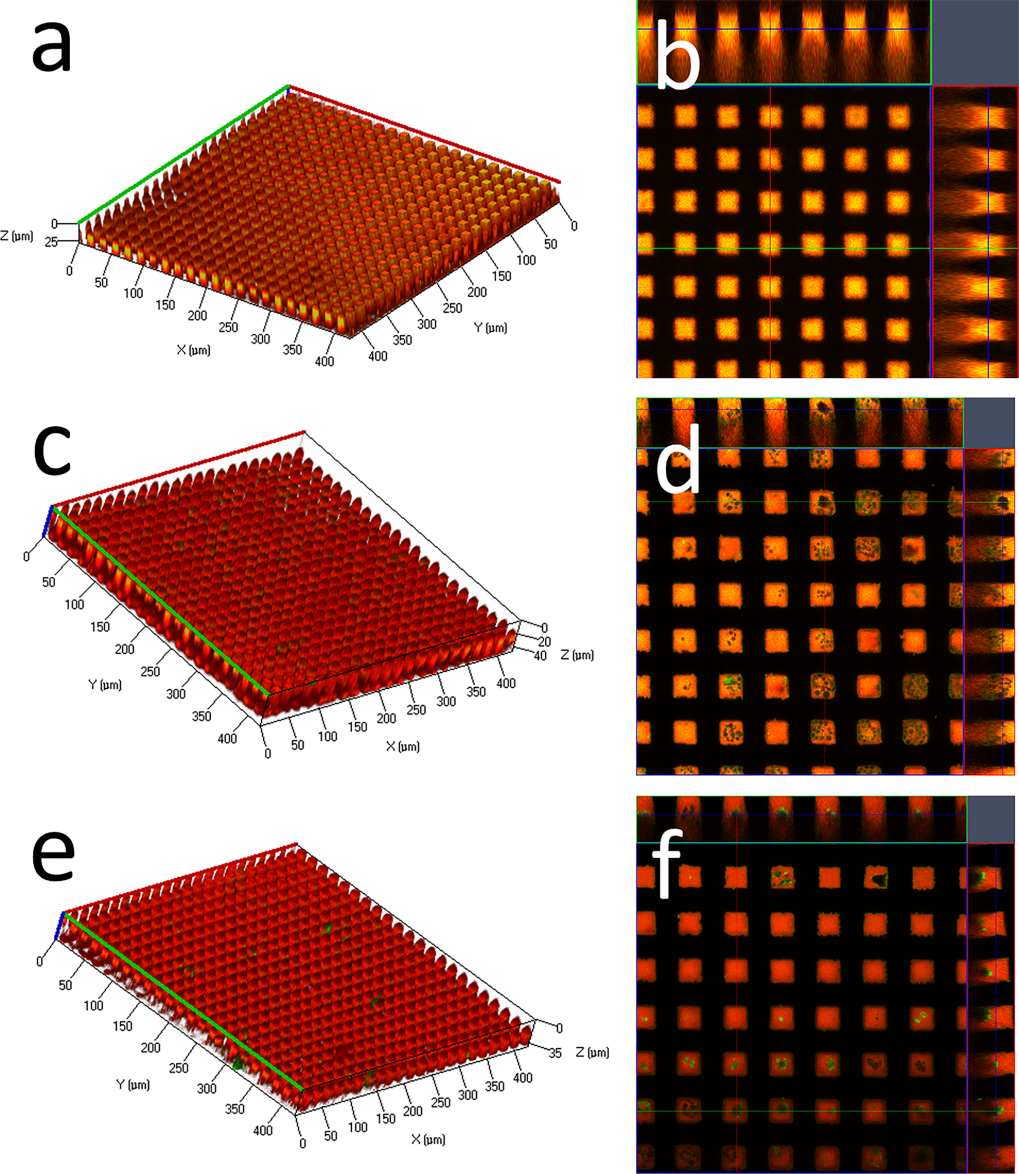
CLSM images and 3D visualization of microchambers incubated for (a, b) 0 days, (c, d) 1 week, and (e, f) 2 weeks in 0.01 M PBS at 37 °C. The PLA was labeled with Nile Red and is shown as red in the images and dexamethasone-FITC is shown in green.

Further sample analysis was carried out using confocal laser scanning microscopy. Figure 6a,b shows that dexamethasone-FITC had been successfully encapsulated in the microchambers made from 2% PLA solution. Nile Red dye was used to label the PLA shell and shown in red. The green color indicates that the sample is filled with the drug inside the microchambers. After 1 week of sample incubation in the PBS (Figure 6c,d), dexamethasone was considerably less present in the microchambers. Some smaller particles of dexamethasone power can be noticed in CLSM images; however, most of the drug has been released and it is in correspondence with release studies. The two-week-old sample shows close to none fluorescent particles of dexamethasone in microchambers (Figure 6e,f). It appears that most of the cargo (labeled dexamethasone) was released from the microchambers as the absence of green color indicates the presence of the drug inside the sample.

**Figure 7:**
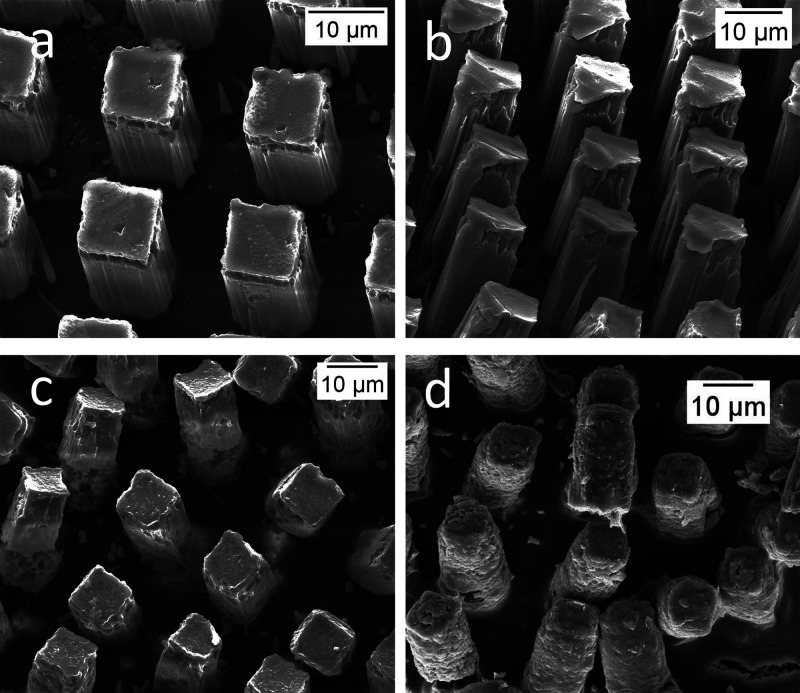
SEM images of microchamber freestanding film after (a) 1 week, (b) 2 weeks, (c) 3 weeks, and (d) 12 weeks of incubation in 0.01 M PBS at 37 °C.

SEM was used for the observation and comparison of microchamber surface morphology during its incubation. As shown in Figure 3d, the surface of the prepared film was smooth and flat with periodic microchambers. Figure 7a shows that after 1 week of incubation in PBS at 37 °C no significant morphological changes can be observed. After 3 weeks, appearance of holes in the microchambers can be noticed. Such changes indicate PLA degradation. The defects were mostly located at the bottom part of the microchambers, where the shell is thinner and most of the drug was located. The corresponding CLSM microscopy images demonstrate that most of the CF dye was released leaving no green color in the microchamber already after 2 weeks. After 12 weeks of incubation, the shape of the microchambers started to change, the sharp edges smoothening with the surface of the microchambers become rough, however, the integrity of the film is still present. According to the results of release and SEM images, it can be concluded that the release mechanism of dexamethasone may be due to its diffusion through the microchamber shell and possibly polymer degradation.

**Figure 8:**
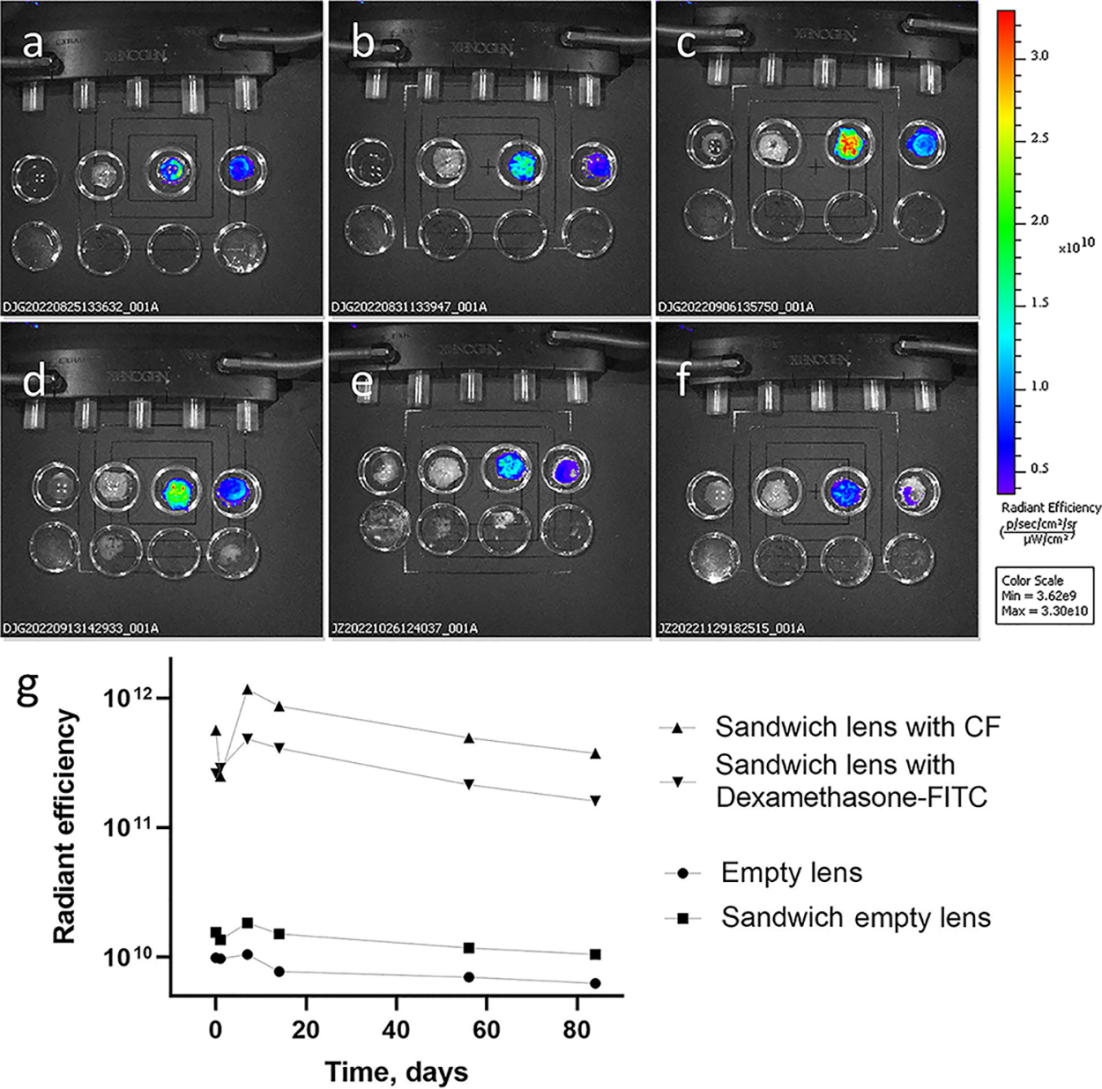
IVIS images of sandwich lenses after (a) 0 days, (b) 1 day, (c) 1 week, (d) 2 weeks, (e) 8 weeks, and (f) 12 weeks of incubation in PBS. In each image from left to right—empty pHEMA lens, sandwich pHEMA lens with an empty microchamber film, sandwich pHEMA lens with a microchamber with encapsulated CF, sandwich pHEMA lens with a microchamber with encapsulated dexamethasone-FITC, and (g) radiance efficiency of samples.

To visualize dexamethasone distribution in the hydrogel lens, an IVIS Fluorescence and Bioluminescence instrument was used. For that, dexamethasone-FITC and CF (as a model fluorescent agent) were encapsulated into microchambers and subsequently into the lens. Empty hydrogel lens and sandwich hydrogel with empty microchambers were used as controls and are shown in Figure 8 as well. The radiance efficiency of samples was measured during the course of the study and is shown in Figure 8g.

In Figure 8a, it can be seen that dexamethasone and CF on the first day were located only in the microchamber region as fluorescence is seen in a doughnut shape indicating dexamethasone and CF distribution. On the next day, the bright fluorescent signal could be found in a bigger area (Figure 8b). After 1 week of incubation, a cargo is spread in the lens and shows the highest amount of fluorescence during the experiment (Figure 8c). The second week of incubation leads to a decrease in the fluorescence signal from samples (Figure 8d), which is consistent with release studies. The signal from the dexamethasone-FITC encapsulated lens can be detected for over 12 weeks of the experiment, leaving only a small spot at the end (Figure 8e,f). Therefore, dexamethasone-FITC sandwich lens with PLA microchambers can release the drug for 12 weeks, the release rate most likely can be increased by using faster degradable polymer such as PLGA or PCL, and it also can be adjusted with the PLA molecular weight and thickness of the polymer shell. Previously, it was shown that such polymers tend to release cargo faster,^21^ which could be preferable for contact lens drug delivery systems.

**Figure 9:**
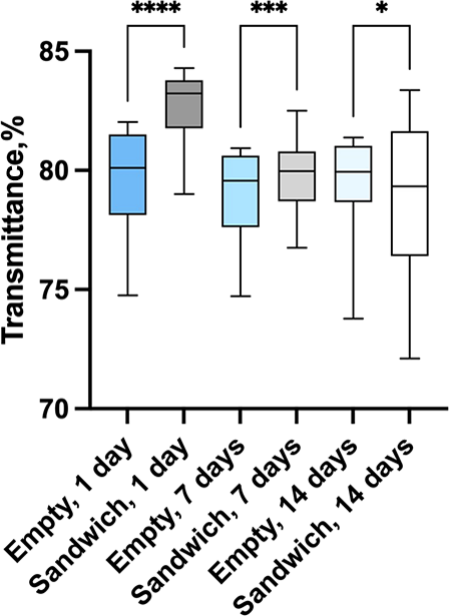
Optical transmittance of the pHEMA lenses with and sandwich pHEMA lenses incubated for 1, 7, and 14 days in PBS solution, *p > 0.1, **p = 0.0001, and ****p < 0.0001.

To understand whether the release of dexamethasone into the hydrogel affects light transmittance through the lens, light transmittance was measured, and the results are shown in Figure 9. The average light transmission in the visible light range (390–700 nm) through the sandwich lenses did not show any trend during the course of the 2 weeks release studies. The light transmission was around the same values with changes mostly due to defects in lenses which were not caused by the presence of the microchamber film and dexamethasone. The results are in correspondence with previous studies as it was also shown previously in the literature that dexamethasone does not affect hydrogel lens light transmittance.^37,38^

## Conclusions

In this work, we demonstrated proof-of-concept of a sandwich contact lens hydrogel drug delivery system. The results show that sandwich lenses with microchambers made via a soft lithography approach provide steady zero-order release of dexamethasone. The release can be controlled further with the size of the film and the shape of the microchambers. This approach can be applied to various types of drugs as there was almost no contact with a solvent. The possible combinations of polymers and ophthalmological drugs enable the potential microchamber application in drug-eluting contact lens development, including multicomponent drug delivery with time-specific drug release for each particular drug in the system.

Nevertheless, multilayer contact lenses face some problems, such as a multistep manufacturing process, degradation of the polymer during storage in the packaging solution, premature release of the drug into the contact lens, and reduced ion and oxygen permeability, all of which may limit the application of this technology in industrial production. One of the important issues in the development of contact lenses is storage. Ordinary contact lenses are often stored for many months at room temperature in an aqueous solution. Thus, the components of the contact lens must not degrade during this time and release the drug. One way to solve this problem is to store the lenses in an environment containing a concentration of the drug sufficient to stop the leakage of the drug from the lens. A simpler option could be to store the lenses in a dehydrated state to avoid drug leakage and polymer degradation.^14^

The developed technology can be separated into two stages—microchamber preparation and hydrogel sandwich lens preparation. The first stage would be the most time-consuming and complicated, which requires several steps—wafer preparation, stamp preparation, and microchamber preparation. Then several optimizations can be introduced to simplify the process. For example, instead of a costly silica wafer, the laser ablation method^42^ can be used to skip that necessity. Another possible change that could be introduced for the delivery of macromolecules is changing the microchamber composition to polyelectrolyte multilayers,^24,30,43^ which can be easily functionalized to achieve desired properties.^44,45^

## Supporting Information Available

Additional results of the smaller microchamber film and SEM images of the free-standing microchamber film (PDF)
