# Supplementary material for: Drug-Eluting Sandwich Hydrogel Lenses Based on Microchamber Film Drug Encapsulation
Source: ACS Nanosci Au. 2023 Apr 5;3(3):256–65. doi: 10.1021/acsnanoscienceau.2c00066 (PMC10288497; doi:10.1021/acsnanoscienceau.2c00066)
Supplement: Supplementary file 1 — ng2c00066_si_001.pdf [file ng2c00066_si_001.pdf]

Supporting information

Drug-eluting sandwich hydrogel lenses based on microchamber film drug encapsulation

Valeriya Kudryavtseva[1,2], Mariana Otero[1], Jiaxin Zhang[3], Anton Bukatin[4,5], David Gould[3], Gleb B. Sukhorukov[1,6,7]*

[1] School of Engineering and Materials Science, Queen Mary University of London, London, E1 4NS, United Kingdom
[2] National Research Tomsk Polytechnic University, 30 Lenin Avenue, Tomsk, 634050, Russian Federation
[3] Biochemical Pharmacology, William Harvey Research Institute, Queen Mary University of London, London EC1M 6BQ, United Kingdom
[4]Alferov Saint Petersburg National Research Academic University of the Russian Academy of Sciences, 8/3A Khlopina str, Saint Petersburg, 194021, Russia
[5] Institute for Analytical Instrumentation of the Russian Academy of Sciences, 31-33 A, Ivana Chernykh str., Saint Petersburg, 198095, Russia
[6] Skolkovo Institute of Science and Technology, Bolshoy Boulevard 30, bld. 1, Moscow, 121205, Russian Federation
[7] Siberian State Medical University, Moskovskiy Trakt, 2, Tomsk, 634050, Russia

[*] Corresponding author

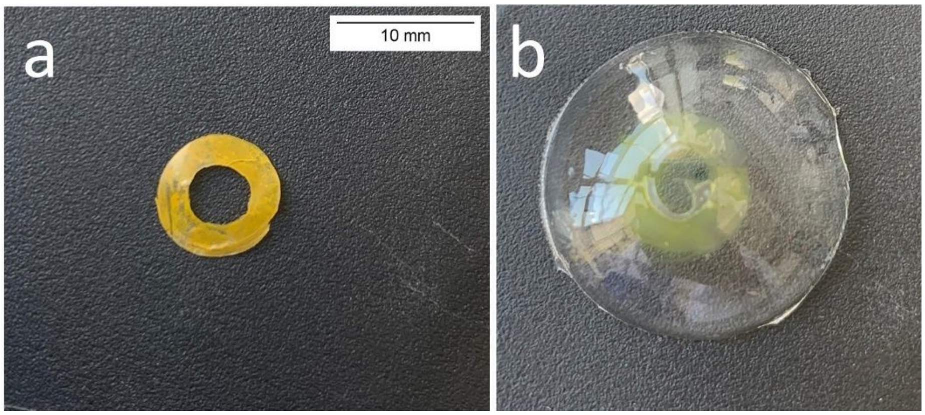

Supporting Figure 1. Photo of a) microchamber film filled with carboxyfluorescein with 9 mm diameter and 5 mm diameter aperture, b) pHEMA sandwich with microchamber film filled with 5(6)-carboxyfluorescein.

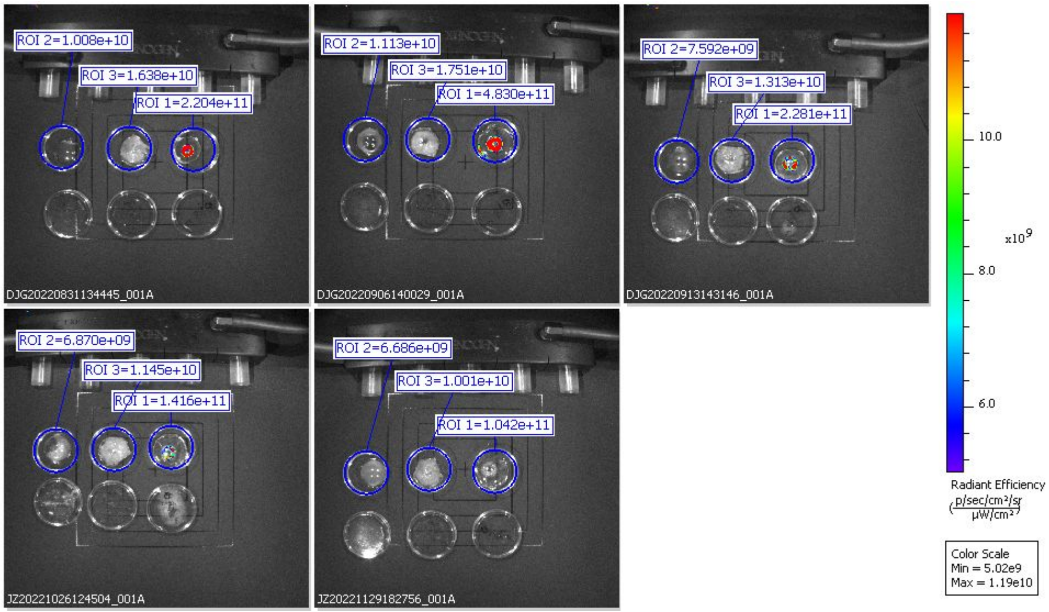

Supporting Figure 2. IVIS images of sandwich lenses after a) 1 day, b) 1 week, c) 2 weeks, d) 8 weeks and e) 12 weeks of incubation in PBS. In each image from left to right – empty pHEMA lens, sandwich pHEMA lens with empty microchamber film, sandwich pHEMA lens with microchamber with encapsulated DEX-FITC.

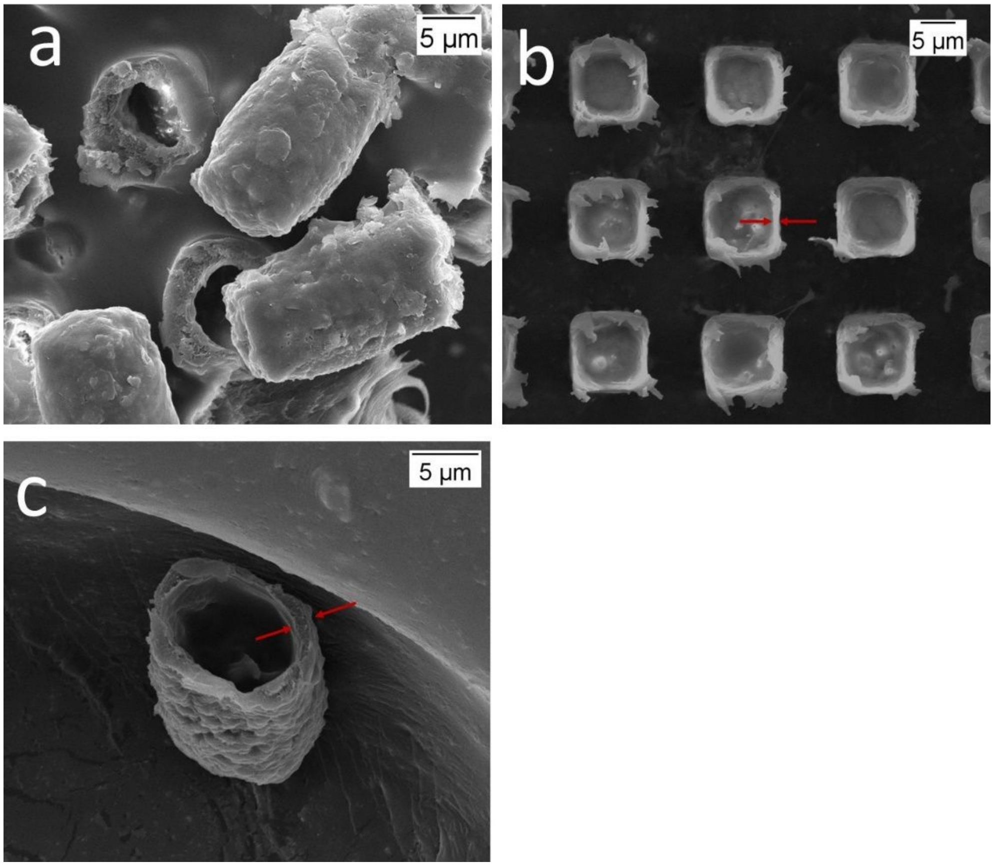

Supporting Figure 3. SEM images of broken microchambers.

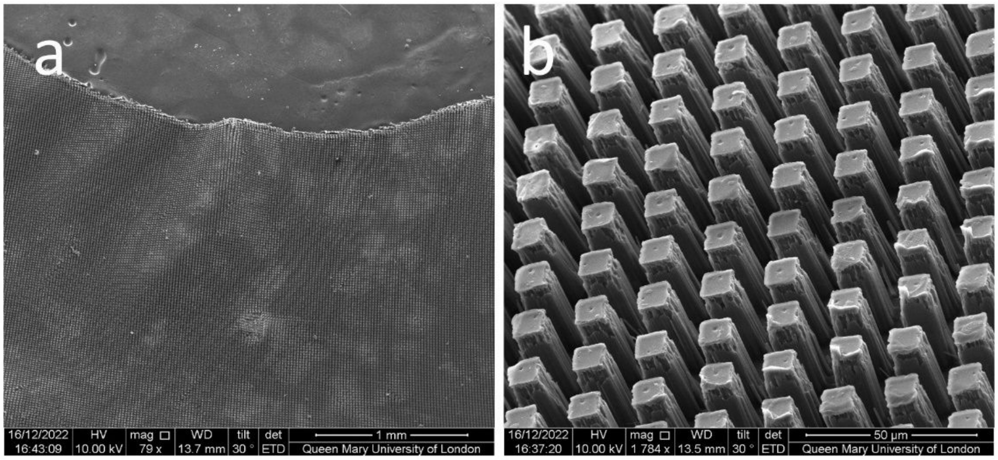

Supporting Figure 4. SEM images of free standing microchamber film.
